# Antimicrobial Strategies in the Era of Resistance: It Is Too Early to Give Up Antibiotic Therapy

**DOI:** 10.3390/ijms27188331

**Published:** 2026-09-19

**Authors:** Jennifer M. Soares, Fernanda Alves, Koteswara Rao Yerra, Thalita H. N. Lima, Nadim Younes, Kate C. Blanco, Vanderlei S. Bagnato

**Affiliations:** 1 Biomedical Engineering, Texas A&M University, College Station, TX 77843, USA; ykrao@tamu.edu (K.R.Y.); thalita.lima@usp.br (T.H.N.L.); nadyounes@tamu.edu (N.Y.); bagnatovs@tamu.edu (V.S.B.); 2 São Carlos Institute of Physics, University of São Paulo, São Carlos 13566-590, SP, Brazil; kateblanco@ifsc.usp.br; 3 CPRIT Scholar in Cancer Research, Texas A&M University, College Station, TX 77843, USA

**Keywords:** antibiotic resistance, alternative therapies, photosensitizers, photodynamic therapy

## Abstract

The alarming rise of antimicrobial resistance constitutes a critical global health challenge, threatening the efficacy of conventional antibiotic therapies. Despite this crisis, abandoning antibiotics is premature; instead, a deliberate integration of traditional and emerging antimicrobial approaches is urgently required. The worldwide proliferation of resistant pathogens reflects decades of monotherapy, inadequate stewardship, and the persistent expectation that each new drug class would resolve resistance crises. This review provides a comprehensive overview of the fundamental mechanisms underlying antibiotic action and bacterial resistance, including reduced membrane permeability, efflux pump activity, target modification, and enzymatic inactivation. Building on this foundation, a broad spectrum of innovative antimicrobial strategies designed to complement or enhance antibiotic efficacy was critically examined. These approaches include natural products with intrinsic antimicrobial properties, immunotherapy to modulate host defenses, and antimicrobial photodynamic therapy (aPDT), which utilizes light-activated photosensitizers to generate reactive oxygen species and achieve localized microbial destruction. Notably, aPDT can overcome bacterial resistance, thereby restoring antibiotic effectiveness. Light delivery to infection sites is feasible across nearly all anatomical locations, whether by direct application, fiber optics, or endoscopic methods. Additional promising modalities, such as bacteriophage therapy, metallic and metal-oxide nanoparticles, and antimicrobial peptides (AMPs), are discussed with respect to their mechanisms, advantages, and translational potential. Collectively, these strategies represent a paradigm shift from antibiotic replacement to antibiotic revival and potentiation. By integrating multidisciplinary approaches, it is possible to extend the lifespan of current antibiotics while developing more robust, resistance-resilient therapies. The evidence presented confirms that antibiotic therapy remains an essential component of antimicrobial treatment, provided that it is supported by innovative, complementary technologies. Abandoning antibiotic therapy at this stage is unwarranted.

## 1. Introduction

**Figure 1:**
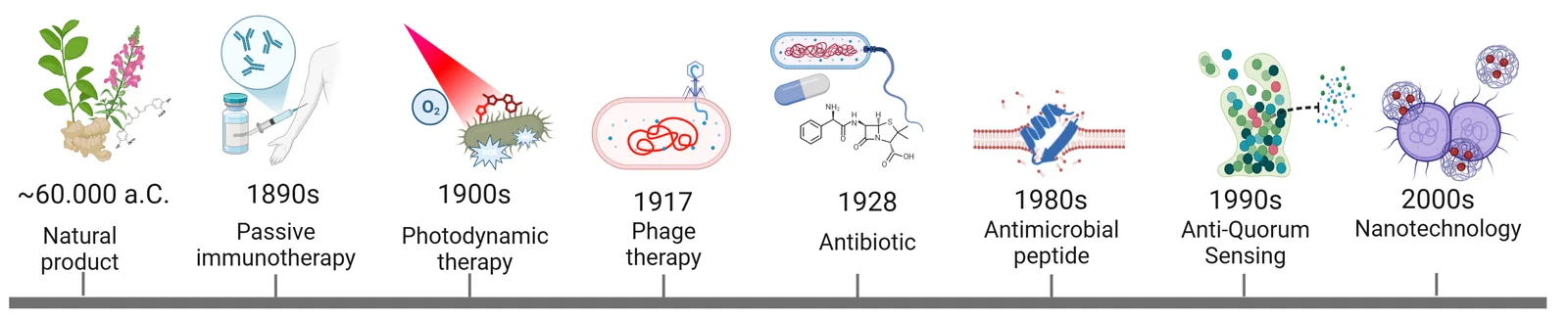
Historical evolution of antimicrobial strategies. The timeline illustrates the transition from the empirical use of natural products in prehistory (~60,000 BC) to modern molecular therapies. Notable milestones include passive immunotherapy (1890) and photodynamic therapy (1900), followed by the discovery of phage therapy (1917) and the era of antibiotics (1940). Contemporary approaches, such as antimicrobial peptides (1980), quorum-sensing inhibitors (1990), and nanotechnology (2000), reflect efforts to develop new mechanisms to overcome global bacterial resistance. Created in Biorender. Jennifer Soares. (2026). https://www.biorender.com.

Antibiotics have revolutionized modern medicine and remain indispensable for the treatment of bacterial infections [1]. Since their introduction, antimicrobial agents have been a key factor in the increase in global life expectancy [2]. However, the selective pressure exerted by their widespread and often indiscriminate use has led to the emergence and dissemination of bacterial strains resistant to multiple classes of antimicrobial agents [3]. In this context, resistant bacteria, along with resistant parasites and viruses, cease to respond to treatment through a broad range of mechanisms, classified as either intrinsic or acquired [4]. The progressive emergence of resistant strains has become a major global health concern. The World Health Organization (WHO) has declared antimicrobial-resistant microorganisms (AMR) among the top ten global public health threats [5,6]. AMR was associated with an estimated 4.95 million deaths globally in 2019 [7,8]. According to the O’Neill report [9], if no action is taken, by 2050, there will be 10 million deaths per year attributable to infections caused by resistant microorganisms. The One Health concept recognizes the interconnected nature of human, animal, plant, and environmental health. AMR is a major One Health challenge, as antimicrobial misuse in healthcare, veterinary medicine, and agriculture, together with environmental contamination, promotes the emergence and spread of resistant microorganisms. Therefore, coordinated surveillance, antimicrobial stewardship, and cross-sectoral collaboration are essential to mitigate AMR [6]. The historical evolution of antimicrobial strategies is shown in Figure 1.

In this scenario, several alternative therapeutic strategies have been proposed, including (i) natural antimicrobials, (ii) immunotherapy, (iii) antimicrobial photodynamic therapy (aPDT), (iv) phage therapy, (v) nanoparticles, (vi) antimicrobial peptides, and (vii) quorum-sensing modulation for virulence regulation. Among these, aPDT warrants particular attention in this review. Antimicrobial photodynamic therapy is based on the activation of a photosensitizer with specific wavelengths of light to generate reactive oxygen species that simultaneously damage multiple microbial targets, it operates through a mechanism that is broadly applicable, and based on current experimental evidence, appears less prone to circumvention by bacteria than single-target agents, although long-term evolutionary studies remain limited [10]. This review examines how each of these strategies can help mitigate antimicrobial resistance and provides a critical comparative analysis of their mechanisms, advantages, and limitations, positioning aPDT both as an independent strategy and as a platform that can strengthen the therapeutic potential of the other approaches reviewed. As a necessary foundation, the following sections outline the primary antibiotic classes and their mechanisms of action, followed by the principal resistance mechanisms that currently undermine them, a context essential for evaluating each of the strategies discussed in this review.

### 1.1. Antibiotic Classes and Mechanisms of Action

Antibiotics are antimicrobial agents produced naturally by microorganisms or synthesized chemically to inhibit the growth of, or eliminate, pathogenic microorganisms [11]. Antibiotics are commonly categorized according to their chemical structures and mechanisms of action. Major antibiotic classes include β-lactams, macrolides, tetracyclines, aminoglycosides, fluoroquinolones, sulfonamides, glycopeptides, and oxazolidinones [12]. Among these, β-lactam antibiotics are among the most widely used classes and include penicillins, cephalosporins, carbapenems, and monobactams [13]. Penicillins, derived from Penicillium species, include amoxicillin, ampicillin, carbenicillin, nafcillin, and penicillin V, whereas cephalosporins include cefadroxil, cefprozil, ceftibuten, and cephalothin. Both subclasses share a β-lactam ring, which is essential for antibacterial activity [13]. Cephalosporins are further classified into generations based on their antimicrobial spectrum. First-generation cephalosporins primarily target Gram-positive bacteria, whereas second- and third-generation agents exhibit expanded Gram-negative coverage. Fourth-generation cephalosporins, such as cefepime, exhibit enhanced stability against β-lactamases and improved penetration across the blood–brain barrier, making them valuable for treating severe infections, including meningitis and encephalitis [13].

Other structurally distinct classes include macrolides, tetracyclines, aminoglycosides, fluoroquinolones, glycopeptides, and oxazolidinones. Macrolides, such as erythromycin and azithromycin, contain a macrocyclic lactone ring and are largely derived from Streptomyces species [12]. Tetracyclines possess a four-ring core structure, while fluoroquinolones are fully synthetic agents characterized by a quinoline scaffold with fluorine substitution that enhances antibacterial efficacy [12]. Glycopeptides, including vancomycin and teicoplanin, are composed of glycosylated peptide frameworks and exhibit potent activity against Gram-positive bacteria. Oxazolidinones, represented by linezolid and tedizolid, are synthetic antibiotics developed to combat multidrug-resistant Gram-positive pathogens [12].

Antibiotics exert their antibacterial effects through interference with essential cellular processes. Based on their mechanism of action, they can be broadly classified into five major groups: (i) inhibitors of cell wall biosynthesis, (ii) inhibitors of protein synthesis, (iii) inhibitors of nucleic acid synthesis, (iv) inhibitors of metabolic pathways, and (v) inhibitors of cell membrane function [14]. Inhibition of bacterial cell wall biosynthesis is one of the most successful antibacterial strategies, as the cell wall is essential for maintaining bacterial integrity, morphology, and osmotic stability [15,16]. The bacterial cell wall consists primarily of peptidoglycan, a mesh-like polymer composed of alternating N-acetylglucosamine (NAG) and N-acetylmuramic acid (NAM) residues connected by β-glycosidic linkages [15,16]. Disruption of peptidoglycan synthesis weakens the cell wall, leading to osmotic instability, autolysis, and ultimately cell death. β-Lactam antibiotics inhibit penicillin-binding proteins (PBPs), which catalyze the cross-linking of peptidoglycan strands during cell wall synthesis. In contrast, glycopeptides such as vancomycin bind to the D-alanyl-D-alanine termini of peptidoglycan precursors, thereby preventing proper cell wall assembly [15,16]. These agents are widely used to treat respiratory tract infections, urinary tract infections, skin infections, and systemic bacterial diseases [15,16].

Another major target is bacterial protein biosynthesis, which occurs on the 70S ribosome, composed of 30S and 50S subunits. Inhibition of protein synthesis ultimately disrupts essential cellular functions, leading to growth arrest or cell death [17]. Tetracyclines and aminoglycosides bind primarily to the 30S subunit, interfering with aminoacyl-tRNA attachment and translational fidelity [17]. In contrast, macrolides, chloramphenicol, and oxazolidinones target the 50S subunit and inhibit peptide bond formation or translocation. These agents are commonly used against severe Gram-negative infections, sepsis, endocarditis, and complicated urinary tract infections [17].

Antibiotics that interfere with nucleic acid synthesis disrupt DNA replication and transcription by targeting bacterial topoisomerases or RNA polymerase [18]. DNA gyrase and topoisomerase IV are essential enzymes that maintain DNA topology during replication [18]. Fluoroquinolones inhibit these enzymes, thereby preventing DNA replication and chromosome segregation. Metronidazole, a nitroimidazole prodrug, generates cytotoxic intermediates under anaerobic conditions that damage bacterial DNA and is widely used against anaerobic bacteria and protozoal pathogens [18].

Nitrofurantoin similarly induces oxidative damage to bacterial nucleic acids and proteins and is commonly employed to treat urinary tract infections. Rifamycins, including rifampicin and rifapentine, inhibit bacterial RNA polymerase and play a central role in the treatment of tuberculosis and other mycobacterial infections [18]. Interference with bacterial metabolic pathways represents another important antibacterial mechanism [18]. Folate metabolism is essential for the synthesis of nucleotides required for DNA replication and cell division. Sulfonamides are structural analogs of p-aminobenzoic acid and inhibit dihydropteroate synthase, thereby blocking folate biosynthesis. Diaminopyrimidines such as trimethoprim inhibit dihydrofolate reductase (DHFR), preventing the formation of tetrahydrofolate [18]. Combined formulations, such as co-trimoxazole, exhibit synergistic activity and are widely used to treat urinary tract, respiratory, and gastrointestinal infections [18].

Finally, several antibiotics target the bacterial cell membrane, disrupting its integrity and permeability, resulting in leakage of intracellular contents and rapid cell lysis [19]. Polymyxins, including Polymyxin B and colistin (Polymyxin E), are cationic amphipathic peptides that interact with lipopolysaccharides in the outer membrane of Gram-negative bacteria [19]. Daptomycin, another membrane-active antibiotic, depolarizes the cytoplasmic membrane of Gram-positive bacteria, thereby inhibiting essential cellular processes [19]. Collectively, these agents target multiple essential bacterial processes, including cell wall biosynthesis, protein synthesis, nucleic acid replication, metabolic pathways, and membrane integrity.

### 1.2. Mechanisms of Bacterial Resistance

**Figure 2:**
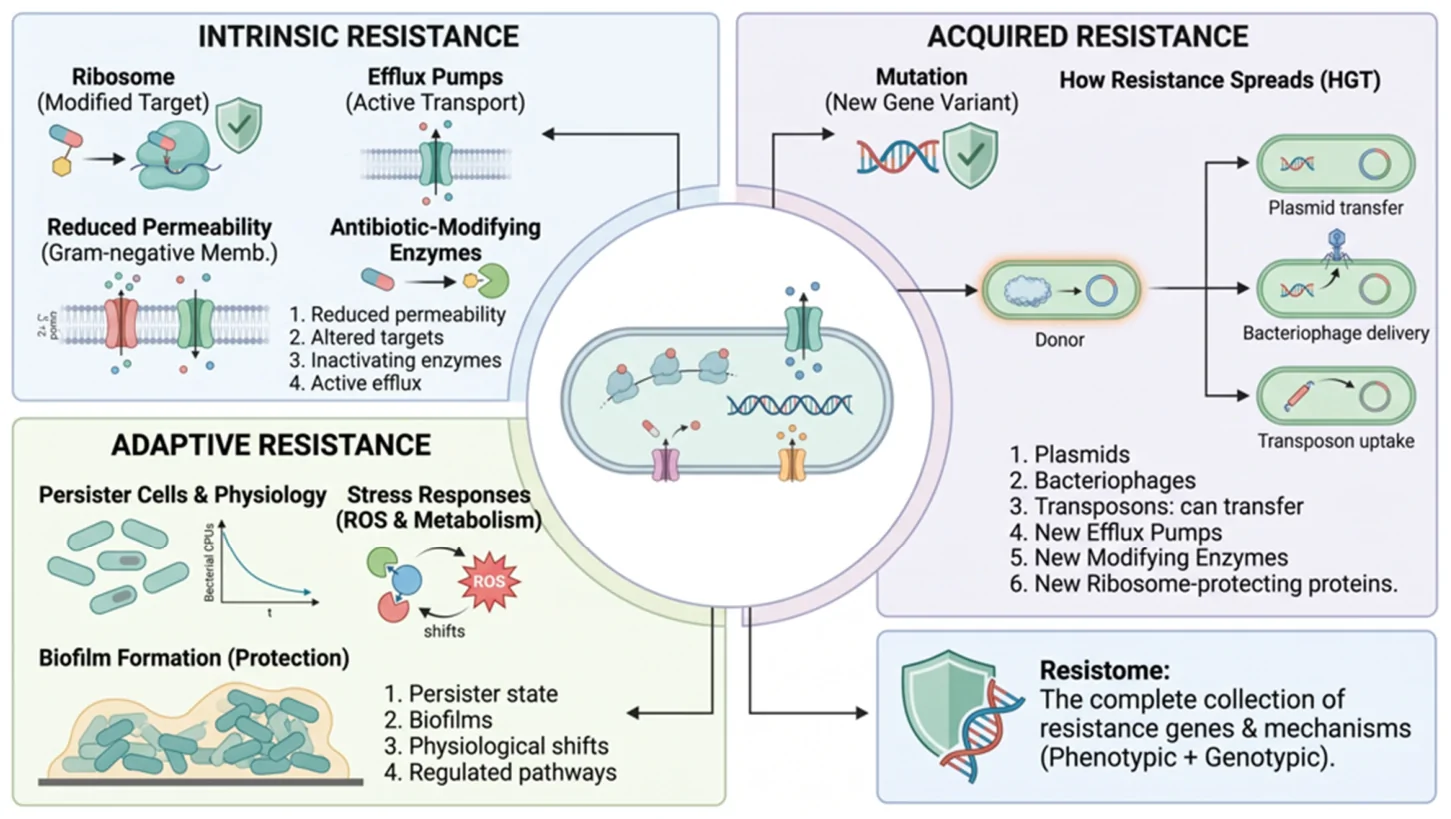
Classification of bacterial antibiotic resistance mechanisms. The central bacterial cell illustrates the three main resistance types within the resistome: Intrinsic: including efflux pumps and inactivating enzymes.Adaptive:representing temporary responses to environmental stress such as biofilm formation; and Acquired: involving genetic changes through target mutations or horizontal gene transfer (HGT). The arrows in the icons are part of the graphic design style and do not show extra data or direction.Created in Biorender. Jennifer Soares. (2026). https://www.biorender.com.

AMR is an emerging public health crisis that research groups worldwide are actively working to address. The primary driver of this phenomenon is the inappropriate use of antibiotics, which selects for bacteria that develop resistance and defense mechanisms against currently available antimicrobial agents [3]. Understanding these mechanisms and the cascades that lead to therapeutic failure is essential for developing countermeasures that can inhibit or reverse resistance. The two major categories of resistance are intrinsic and acquired [4]. Intrinsic resistance is a constitutively expressed trait in a given bacterial species that does not require prior antibiotic exposure; it is characterized by structural features such as outer membrane permeability barriers and constitutively active efflux pumps [20]. In contrast, acquired resistance arises through horizontal gene transfer or genetic mutations induced by environmental stressors such as UV radiation or nutrient deprivation. These changes can result in various resistance mechanisms: (i) alteration in the permeability of the outer membrane of Gram-negative bacteria [4], (ii) increased expression of efflux pumps capable of expelling antimicrobials [21], (iii) modifications in the target of the antimicrobial [4], and (iv) production of enzymes capable of neutralizing the drug [22]. In this way, these changes can affect both the uptake of antimicrobial agents and their efficiency in neutralizing microorganisms [23]. The mechanism of bacterial resistance is summarized in Figure 2.

#### 1.2.1. Outer Membrane Permeability

In Gram-negative bacteria, the outer membrane serves as a primary barrier limiting antibiotic uptake before the drug can reach its intended target. Entry occurs through two main routes: general diffusion porins (OMPs) for hydrophilic molecules, and a lipid-mediated route for hydrophobic compounds [24]. Certain organisms have evolved structurally distinct envelopes that inherently restrict drug access; Mycobacteria, for example, possess a highly lipid-rich outer membrane that limits the entry of hydrophilic drugs, while bacteria lacking a cell wall, such as Mycoplasma, are intrinsically resistant to all cell wall-targeting agents, including β-lactams and glycopeptides [4]. These permeability-based strategies represent a passive yet effective layer of defense that synergizes with other mechanisms, such as efflux pumps, to confer multidrug resistance [4,24].

#### 1.2.2. Efflux Pumps

Efflux pumps are membrane-associated transport proteins that actively expel antibiotics and other toxic substrates from the cell before they can reach their intended targets [21]. The substrate specificity of these systems is broad, meaning that a single pump can confer resistance to structurally unrelated drug classes [20]. Efflux pumps are classified into five major families based on structure and energy source: ABC, MATE, SMR, MFS, and RND [4]. Among these, the RND family is particularly significant clinically, as it is primarily responsible for both intrinsic and acquired multidrug resistance in Gram-negative bacteria [20,25]. RND pumps span the cytoplasmic membrane, periplasm, and outer membrane, enabling the direct extrusion of substrates from within the cell [20]. In *Pseudomonas aeruginosa*, for example, the RND-type system MexAB-OprM confers intrinsic resistance to multiple antibiotic classes, including β-lactams and fluoroquinolones, two of the most critically relied-upon treatments for Gram-negative infections [20].

#### 1.2.3. Alteration of Antibiotic Targets

Bacteria can also develop resistance through the enzymatic modification of antibiotic target sites, reducing or completely preventing drug binding. This has emerged as an important mechanism affecting multiple classes of antibiotics [26]. A prominent example involves penicillin-binding proteins (PBPs), which are essential for peptidoglycan synthesis in the bacterial cell wall. Alterations in the number or structure of PBPs decrease the binding affinity of β-lactam antibiotics [4], including ceftazidime and gentamicin. Target modification can also occur through genetic mutations affecting ribosomal subunits or enzymes involved in DNA replication. Mutations in ribosomal components impair antibiotic binding, while mutations in DNA-related enzymes reduce susceptibility to fluoroquinolones, such as ciprofloxacin and delafloxacin [4].

#### 1.2.4. Enzymatic Inactivation

Some bacterial enzymatic mechanisms inactivate antibiotics through direct drug degradation or chemical modification. A major example is the class of β-lactamases, a diverse group of enzymes that hydrolyze β-lactam antibiotics, rendering them ineffective [4]. Additionally, certain enzymes transfer chemical groups, such as acetyl, phosphate, or adenyl moieties, directly onto antibiotic molecules. Acetylation represents the most widespread modification mechanism, affecting multiple antibiotic classes, while phosphorylation and adenylation primarily target aminoglycosides [4]. Other resistance-associated enzymes can deactivate prodrug antibiotics through metabolic modification, further contributing to drug inactivation [27].

## 2. Approaches

### 2.1. Antimicrobial Natural Products

Natural products derived from diverse biological sources, including plants, microorganisms, and animals, represent a highly promising class of agents for combating microbial infections [28]. Historically, these compounds served as a cornerstone in the discovery and development of antimicrobial therapeutics. Traditional remedies such as garlic, honey, ginger, and various essential oils are among the earliest known natural antibiotics, with documented use dating back to ancient civilizations. Notably, many of these natural products demonstrate efficacy against multidrug-resistant (MDR) pathogens, primarily through mechanisms such as the disruption of microbial membranes and interference with essential metabolic processes [29]. Medicinal plants, including herbs and plant-derived materials, have long been used in traditional medicine to treat various infectious diseases. Their therapeutic potential is largely attributed to phytochemicals, structurally diverse secondary metabolites that exhibit potent antimicrobial properties [29]. These compounds exert their effects through multiple mechanisms, including direct bactericidal activity, disruption of key cellular functions, and synergistic enhancement of conventional antibiotics, thereby helping to overcome microbial resistance. Major classes of antimicrobial phytochemicals include flavonoids, alkaloids, terpenes, and saponins, among others (Table 1).

Among these, flavonoids are particularly well-studied due to their broad-spectrum antimicrobial activity. Widely present in plant-based foods and herbal medicines, flavonoids have demonstrated significant efficacy against drug-resistant pathogens, including methicillin-resistant *Staphylococcus aureus* (MRSA) and vancomycin-resistant *Enterococci* (VRE) (Table 1). Their mechanism of action includes the inhibition of bacterial cell wall synthesis, leading to cell lysis and death. Additionally, flavonoids interfere with biofilm formation, a key factor contributing to the persistence and resistance of MDR infections, by disrupting the extracellular matrix and bacterial communication systems. Furthermore, they have been shown to inhibit efflux pumps (Table 1). Terpenoids constitute another major class of natural compounds with diverse biological activities. Classified based on the number of isoprene units (e.g., monoterpenes, sesquiterpenes, diterpenes, triterpenoids), terpenoids exert antimicrobial effects primarily through the disruption of microbial cell membranes and structural integrity. Similarly, alkaloids, nitrogen-containing heterocyclic compounds found across plants, animals, and microorganisms, represent attractive candidates for drug development (Table 1).

Plant-derived resins represent another important category of antimicrobial natural products. These complex mixtures of secondary metabolites, including phenolics, terpenes, and alkaloids, serve as protective agents in plants against pathogenic invasion [30]. Substances such as propolis and myrrh have been used medicinally for centuries due to their broad-spectrum antimicrobial activity. Propolis, a resinous material produced by honeybees, contains a rich composition of flavonoids, phenolic acids, waxes, vitamins, and minerals, contributing to its diverse pharmacological properties [30]. Myrrh, derived from *Commiphora molmol*, exhibits a unique ability to preferentially target non-growing bacterial cells, including *E. coli* and *S. aureus*, highlighting its potential in treating persistent infections. In recent years, fungi, particularly edible mushrooms such as *Morchella conica* and *M. esculenta*, have emerged as promising sources of antimicrobial compounds. Studies have demonstrated that extracts from these species exhibit inhibitory activity against MRSA, suggesting their potential as novel antibacterial drug candidates [30].

Essential oils (EOs), volatile mixtures derived from plants, are also well-recognized for their antimicrobial properties [31]. Oils such as tea tree, oregano, thyme, cinnamon, and eucalyptus exert their effects primarily by disrupting microbial cell membranes. Their lipophilic nature facilitates increased membrane permeability, leading to the leakage of intracellular components, structural damage, and ultimately cell death [31]. In addition, EOs can inhibit biofilm formation, suppress efflux pump activity, and interfere with cytoplasmic functions, contributing to both bactericidal and bacteriostatic effects. Microorganisms themselves represent a vast and largely untapped reservoir of bioactive compounds with significant antimicrobial potential. Both bacteria and fungi produce a wide array of antimicrobial substances, including bacteriocins, antibiotics, and biosurfactants [31]. These compounds act through diverse mechanisms, including the inhibition of cell wall synthesis, disruption of membrane integrity, interference with protein synthesis, and the modulation of metabolic pathways. Notable examples include cyclic peptides (e.g., mathiapeptide A, destotamide B, marfomycins A, B, and C), spirotetronate polyketides (e.g., abyssomycin C, lobophorins F and H), as well as alkaloid and sesquiterpene derivatives such as arboxamycin and mafuraquinocins A and D (Table 1).

Given the extensive body of literature in this field, the examples presented here are intended to provide a representative overview of natural antimicrobial agents and their mechanisms of action.

**Table 1:** Antimicrobial properties of various natural agents and compounds.

Agent/Active Compound	Source/Scientific name	Activity/Mechanism	Reference
Antimicrobials from Plants			
Black pepper/piperine, phenolics, terpenoids	*Piper nigrum*	*S. aureus*, *E. coli*, *S. typhi*, *Proteus* spp.	[32]
Coffee Cascara	*Coffea arabica*	Inhibits ESKAPE pathogens	[33]
Ginger/gingerols and shogaols	*Zingiber officinale*	Inhibits Gram-positive and Gram-negative bacteria, including MDR isolates	[34]
Honey/polyphenols	*Apis mellifera*, *A. cerana*	*Clostridium botulinum*, etc.	[35]
Flavonoids			
Apigenin	Fruits, vegetables, herbs	Inhibits MSSA and MRSA, *P. oryzae*, *E. caccae*, etc.	[36]
Baicalein	*Scutellaria baicalensis*	Broad-spectrum antimicrobial activity	[37]
Cyanidin-3-O-glucoside	Berries, Cherries	Inhibits *S. aureus*, *E. coli*, *S. typhimurium*, *L. monocytogenes*/Disrupts bacterial cell wall	[38]
Genistein	Soybean, soy products	Inhibits *S. aureus*, *A. hydrophila*	[39]
Luteolin	Edible plants	Inhibits *S. aureus*, *E. coli*, *S. typhimurium*, *E. cloacae*, and MRSA	[40]
Naringenin	Citrus fruits	Inhibits *B. subtilis*, *P. aeruginosa*	[41]
Terpenoids			
Monoterpenoid, Geraniol	*Helichrysum italicum*	Inhibits MDR *E. aerogenes*, *E. coli*, *P. aeruginosa*, and *A. baumannii*	[42]
Sesquiterpenoid, Farnesol	Citronella, Neroli	Fungicide activity against *P. brasiliensis*	[43]
Diterpenoids, Andrographolide	*Andrographis paniculata*	Inhibits *E. coli*, *K. pneumoniae*, and *S. aureus*	[44]
Triterpenoid, ursolic acid	Peels of fruits and leaves of herbs	Inhibits MRSA	[45]
Alkaloids			
Berberine (BBR)	*Coptis chinensis*	Inhibits MRSA	[46]
Nicotine	Tobacco products	Inhibits *S. aureus* and *M. phlei*	[47]
Antimicrobials from Microbial Origins			
Natamycin	Soil bacteria, *S. natalensis*, *S. chattanoogensis*	Inhibits *Fusarium* species	[48]
Lugdunin	*S. lugdunensis*	Inhibits MRS and VRE	[49]
Teixobactin	*Eleftheria terrae*	Inhibits *S. pneumoniae* and *M. tuberculosis*	[50]
Essential Oils			
Citrus/d-limonene	*Citrus sinensis*	Inhibits *S. aureus*, *E. coli*, *P. aeruginosa*	[51]
Thyme/Thymol	*Thymus vulgaris*	Inhibits *S. aureus*, MRSA, *K. pneumoniae*	[52]
Cinnamon/cinnamaldehyde	*C. camphora*, *C. zeylanicum*, *C. cassia*	Inhibits *K. pneumoniae*, *A. baumannii*, *B. cereus*, *H. pylori*, *M. tuberculosis*	[53]

### 2.2. Immunotherapy

Before the advent of antibiotic treatment for infectious diseases, immune-based therapies were already in use. One such approach involved injecting serum collected from recovered patients into individuals with the same illness, a method that conferred passive immunity via antibody transfer and provided temporary protection [53,54,55]. This approach was subsequently applied to treat various diseases, including tetanus, pneumococcal pneumonia, rabies, and Ebola. It remained one of the only strategies for controlling high-risk bacterial infections until the introduction of antibiotics in the 1940s [54]. With the advent of penicillin and other antibiotics, treatment became more advantageous, as it did not require serotype screening and was more industrially scalable [54,55].

Currently, passive immunization is notable for its use of monoclonal antibodies (mAbs), with most studies focusing on cancer therapy, though applicability to bacterial infection control has also been demonstrated [56,57]. These synthetic antibodies act by binding to specific antigens, such as toxins, bacterial surface proteins, or virulence factors, neutralizing them and facilitating their elimination by phagocytic cells, including neutrophils and macrophages [58,59]. One mAb already approved is bezlotoxumab, which reduces recurrent Clostridioides difficile infections by 38% in high-risk cases by neutralizing toxin B. For *S. aureus*, two antibodies are currently under investigation: suvratoxumab, which targets α-toxin and reduced ventilator-associated pneumonia by 32% in evaluated cases, and AR-301, which increased the cure rate by 40% when combined with antibiotics [60,61]. The adoption of mAbs as a standard treatment is hindered by pharmacokinetic limitations, high structural complexity, and production costs. Therapeutic success depends on the precision of the target, which must be highly expressed in the pathogen; otherwise, in addition to therapeutic failure, there is a risk of increased toxicity due to binding to host tissues via Fc receptors on cells such as macrophages and dendritic cells [60,62].

In active immunotherapy, the approach aims to stimulate the host’s own immune system, providing a prolonged response and the development of immunological memory [54,57]. The vaccine technologies employed are varied: live attenuated microorganisms (e.g., BCG for tuberculosis), inactivated preparations (e.g., pertussis vaccine), conjugate vaccines (e.g., PCV20 for *S. pneumoniae*), toxoids (e.g., VLA84 for C. difficile), and mRNA-based vaccines, a more recent development [60,63]. All aim to trigger humoral immunity through the production of specific antibodies, a process dependent on CD4+ helper and CD8+ cytotoxic T lymphocytes, the latter being essential for intracellular pathogens, as well as on the activation of memory B cells [63,64]. The success of vaccines has ensured the eradication or significant control of various infectious diseases, minimizing their severity and transmission, a landmark achievement of modern medicine. However, the development of vaccines against diverse pathogens is complicated by antigenic diversity and serotype variation, making broad-spectrum vaccine design particularly challenging, as seen with pneumococci and meningococci even in conjugate formulations such as PCV20 [64,65].

Other immunotherapy strategies of interest include immune checkpoint modulators, such as PD-1 (Programmed Death-1), a receptor expressed on CD8+ and CD4+ T cells [57,66]. These checkpoints regulate both the intensity and duration of the immune response, preventing hyperactivation that could lead to immunopathology. In murine models of Burkholderia pseudomallei infection, increased expression of PD-1 and PD-L1 has been observed, thereby suppressing the immune response and promoting bacterial persistence [57,67]. Immunotherapy can therefore act by blocking the PD-1/PD-L1 interaction with monoclonal antibodies, reversing the immune exhaustion observed in sepsis, a condition characterized by exacerbated inflammation driven by cytokines such as TNF-α, IL-6, and IFN-γ [68]. In vivo studies have shown that anti-PD-1 therapy did not cause serious adverse effects in sepsis models, but demonstrated potential to exacerbate infection in tuberculosis [57,60,66].

Advances in immunotherapy for controlling bacterial infections have not yet been explored as extensively as in oncology [56,69,70]. Although each approach carries distinct benefits and limitations, one of the main barriers in the microbiological context is the risk of immunopathology and exacerbated inflammation, as well as bacterial genetic diversity, which hinders the development of universally effective therapies [56,68]. The pathogen–host interaction is crucial for activating immune response cascades involving IL-1, TNF-α, and GM-CSF, and modulating these cascades can shift the balance between effective defense and damage to host tissue [55,57]. Addressing these challenges is critical if immunotherapy is to realize its potential as a complement to conventional antimicrobials, and as the following sections discuss, as a future partner to physicochemical approaches that target multiple sites simultaneously.

Despite this therapeutic promise, several limitations currently constrain the clinical translation of immunotherapy against bacterial infections. Much of the mechanistic evidence for immune checkpoint modulation, monoclonal antibody efficacy, and combined photodynamic-immunotherapeutic strategies in this setting remains less extensive than in oncology, and large-scale clinical validation is still scarce. The high production cost and structural complexity of monoclonal antibodies, together with the risk of Fc-receptor-mediated toxicity to host tissue, further restrict widespread clinical adoption [60,62]. Immune checkpoint blockade, while promising in sepsis models, carries the risk of exacerbating latent or chronic infections such as tuberculosis, underscoring the need for careful patient stratification before clinical use [57,60,66]. Finally, because immune-based strategies act on host physiology rather than directly on the pathogen, their efficacy and safety are inherently more variable across patients, particularly in immunocompromised or critically ill individuals, than those of conventional antimicrobials.

### 2.3. Antimicrobial Photodynamic Therapy (aPDT)

Since antiquity, light has been used as a therapeutic agent for various skin diseases, including vitiligo, psoriasis, and wounds [71,72]. However, it was not until 1903 that the scientist Niels Finsen was awarded the Nobel Prize in Physiology or Medicine for his use of phototherapy, specifically ultraviolet light, to treat cutaneous tuberculosis [73]. Almost simultaneously, the first report on the association of light with a photosensitive molecule was published in 1900 by the German researcher Oscar Raab, who accidentally exposed a culture of Paramecium caudatum containing acridine to white light and observed markedly more intense cytotoxic effects when light and acridine were combined [71,72].

Antimicrobial photodynamic therapy (aPDT) and photodynamic inactivation (PDI) are terms used interchangeably to describe the technique pioneered by Raab when applied to microorganisms; however, the underlying mechanism of action remains identical when applied to eukaryotic cells [74,75]. This technique is approved in numerous countries, particularly throughout Europe, for the treatment of cancer, infections, and various other medical conditions [74,75]. Its action is localized and depends on the simultaneous interaction of light, molecular oxygen (O_2_), and a photosensitizer (PS) to trigger damage in both prokaryotic and eukaryotic cells [76,77]. The individual action of the PS or of light at a specific wavelength should not trigger cytotoxic effects in uninfected tissue. By virtue of this mechanism, aPDT exerts a localized effect, thereby reducing the likelihood of adverse effects. Furthermore, it can be applied in conjunction with other conventional clinical procedures without substantially increasing treatment costs due to the use of light-emitting diodes (LEDs) as the irradiation device [76,77].

The initial stage of aPDT is crucial for achieving favorable outcomes, as it involves the uptake of the PS by the target microorganism, a process modulated by electrostatic interactions between the cell wall and the PS [76,78]. The bacterial surface carries a negative charge, thereby favoring binding with cationic PSs [76,78,79]. Gram-positive bacteria are more susceptible to aPDT because their cell walls are more permeable to PSs, containing more pores that facilitate the entry of neutral and negatively charged molecules, despite electrostatic repulsion by the negative charges of their teichoic acids [78,79]. Analogously, lipopolysaccharides confer a negatively charged cell envelope on Gram-negative bacteria; however, due to the presence of an outer membrane, anionic and neutral photosensitizers are poorly internalized by these organisms [78,79].

Following internalization of the PS, the mechanism of action is activated by photon absorption, which promotes an electron from the ground state (S_0_) to the excited singlet state (S_1_). This state is highly likely to undergo a radiationless transition to the excited triplet state (T_1_) via intersystem crossing [78,80]. This transition involves spin inversion of the electron, which cannot return to the S_0_ state without violating the Pauli Exclusion Principle, as it would share the same quantum numbers as the electron already present in that state [77,80]. For this reason, the T_1_ state has a longer lifetime, allowing it to interact with molecular oxygen (O_2_). The PDT process is distinguished from endogenous cellular oxidative processes by its dependence on light absorption. The interaction between the PS and O_2_ can proceed via two types of reactions, designated Type I and Type II [77,80,81]. In the Type I reaction, the PS in the T_1_ state reacts directly with organic substrates within the cell membrane or cellular components through electron transfer, forming radical ions that react with ground-state oxygen to produce reactive oxygen species (ROS) such as hydrogen peroxide (H_2_O_2_), the superoxide anion radical (O_2_^−•^), and the hydroxyl radical (^•^OH) [77,80,81]. In the Type II reaction, the PS in its T_1_ state transfers energy directly to molecular oxygen, exciting it to its highly reactive singlet state. Singlet oxygen (^1^O_2_) reacts with amino acids, proteins, unsaturated lipids, and nucleic acids, thereby promoting cell death via necrosis or apoptosis [77,80,81].

Cellular biomolecules are damaged by ROS generated during PDT, and simultaneous damage at multiple sites leads to cellular dysfunction [76,81]. Among the primary targets are lipids, particularly those of the plasma membrane, which undergo oxidative modifications and a reduction in the degree of unsaturation of their chains, directly disrupting membrane fluidity and organization, both of which are essential for cellular integrity [76,81]. Bacterial proteins are also affected by phototoxicity, triggering disruptions in various metabolic functions [76,81]. Although aPDT was discovered in the early twentieth century, the development of PSs was limited in the initial decades; moreover, with the discovery of penicillin, efforts to combat infectious diseases were directed primarily toward developing new antibiotics [82,83]. However, with the emergence of resistance across multiple antimicrobial classes, the search for alternatives has gained renewed prominence, leading to a resurgence in the discovery of novel PSs [82,83].

#### 2.3.1. Photosensitizers for aPDT

Over recent decades, a wide variety of PSs have been investigated for antimicrobial applications, encompassing synthetic dyes (e.g., phenothiazinium salts, porphyrins, phthalocyanines) and natural compounds (e.g., curcumin, hypericin, riboflavin) [74,84]. The ideal PS for aPDT should exhibit strong absorption at an appropriate therapeutic wavelength, high quantum yield of ROS generation, preferential accumulation in microbial cells over host cells, chemical and photochemical stability, low dark toxicity, and acceptable cost and ease of formulation [84,85]. Among the most widely studied PSs are the phenothiazine dyes methylene blue (MB) and toluidine blue O (TBO), both positively charged and activated by red light in the 630–670 nm range. Their cationic nature promotes electrostatic binding to microbial membranes and facilitates uptake in both Gram-positive, and to a lesser extent, Gram-negative bacteria [85]. MB-mediated aPDT has achieved reductions exceeding 2 log_10_ CFU/mL against multidrug- and extensively drug-resistant *Acinetobacter baumannii*, *Pseudomonas aeruginosa*, and *Klebsiella pneumoniae* in vitro and shows synergistic effects when combined with conventional antibiotics such as gentamicin [86,87,88,89,90]. TBO incorporated into polymeric microparticles has produced >6 log_10_ reductions in *P. aeruginosa* and *S. aureus* within 30–60 min under low-intensity red light and has demonstrated substantial efficacy against *Candida* spp. in both planktonic and biofilm forms. Porphyrins and chlorin derivatives constitute one of the most structurally diverse PS classes, typically characterized by strong absorption bands at ~400 nm (Soret band) and additional Q bands between 500 and 650 nm [86].

Cationic porphyrins display broad-spectrum antimicrobial activity; a tricationic porphycene derivative, for example, produced a >2 log_10_ reduction in MRSA burden in a murine burn infection model at 180 J/cm^2^ [87]. Among the chlorin-type PSs, Photodithazine^®^ (PDZ) has attracted particular interest for its high singlet oxygen yield and potent activity against biofilms. PDZ-mediated aPDT has achieved reductions of up to ~6–7 log_10_ in MRSA biofilms and complete inactivation of planktonic *Candida albicans* after a few treatment cycles; when combined with DNase I to disrupt extracellular DNA, near-complete eradication of *C. albicans* biofilms has been reported [88].

Zinc phthalocyanines (ZnPc) absorb in the red and near-infrared (NIR) region (650–700 nm), enabling deeper tissue penetration compared with shorter-wavelength PSs [91]. Tricationic ZnPc derivatives exhibit minimal bactericidal concentrations below 0.2 µM against MRSA clinical isolates, often outperforming MB, while maintaining acceptable cytocompatibility with human keratinocytes and fibroblasts [92]. Neutral phthalocyanines are less effective against Gram-negative bacteria, but introducing cationic substituents into the macrocycle can significantly enhance antimicrobial performance by improving binding to negatively charged surfaces and facilitating translocation across membranes [85,91]. Among the xanthene dyes, rose bengal (RB), activated by green light (~520 nm), is notable for its exceptionally high singlet oxygen quantum yield. RB has demonstrated efficacy against *S. aureus* and *Candida* spp. at low to moderate concentrations, and recent preclinical and clinical studies support its use as an adjunctive therapy for infectious keratitis [93]. In vitro, RB-mediated aPDT can significantly reduce *C. albicans* viability in both planktonic and biofilm states, with ongoing trials examining optimal dosing and light parameters for ocular infections [93].

Recent advances in aPDT have highlighted the growing relevance of polyphenolic and other naturally derived compounds as alternative PSs. These molecules possess unique photochemical and structural properties that enable efficient light absorption, ROS generation, and simultaneous intrinsic antimicrobial activity. Natural photosensitizers, including curcumin, riboflavin, phycocyanin, chlorophyll derivatives, and various plant-derived extracts, have demonstrated significant antimicrobial efficacy when activated by specific wavelengths of visible light, targeting a broad spectrum of pathogens. One of the key advantages of natural PSs lies in their favorable safety profile, low toxicity, enhanced biocompatibility, and reduced environmental impact, though these depend on the specific compound, dose, formulation, and exposure conditions [94]. A summary of commonly investigated natural photosensitizers, along with their sources, activation wavelengths, and antimicrobial efficacy, is presented in Table 2.

#### 2.3.2. Light Sources and Optical Parameters

The effectiveness of aPDT depends as much on optical parameters as on the choice of PS. Efficient photon delivery to PS molecules at the infection site requires the careful matching of three factors: (i) light wavelength relative to the PS absorption spectrum, (ii) the type of light source employed, and (iii) the penetration depth of the chosen wavelength in biological tissues. Each PS has characteristic absorption maxima, and illumination that does not adequately overlap these peaks results in inefficient triplet-state excitation and limited ROS generation. Therefore, the PS and the light source must be selected as a pair. In practice, two main categories of light sources are used: lasers and LEDs. Lasers emit coherent, monochromatic light at a precise wavelength, providing high energy density and enabling delivery through optical fibers to anatomically challenging sites such as root canals, periodontal pockets, or deep surgical cavities. Their disadvantages include higher cost, potential heat generation at high power levels, and limited portability. LEDs emit non-coherent, spectrally broader light but are considerably cheaper, portable, and well-suited for treating large surface areas, including oral biofilms, skin wounds, and onychomycosis [104]. While lasers may offer superior penetration and focus for deep-seated infections, numerous studies report comparable antimicrobial outcomes with optimally configured LED-based protocols for superficial or moderately deep lesions [105].

An important concept for tissue light delivery is the optical (therapeutic) window, generally defined between ~600 and 950 nm, within which tissue absorption is relatively low and light penetration is maximal. Below ~600 nm, chromophores such as hemoglobin and melanin absorb strongly, limiting penetration to fractions of a millimeter; above ~950 nm, water absorption becomes dominant, converting much of the energy into heat. Within the optical window, light can penetrate from several millimeters to several centimeters, depending on wavelength and tissue composition. NIR wavelengths around 800 nm may penetrate roughly two to three times deeper than blue or violet light near 400 nm [106]. Accordingly, PSs activated by blue or green light (e.g., curcumin, rose bengal) are best suited for superficial infections, whereas red- or NIR-absorbing PSs (e.g., chlorins, phthalocyanines) are more appropriate for deeper or less accessible sites. Importantly, penetration depth is governed primarily by wavelength, not by irradiance; simply increasing light power does not substantially increase the depth reached.

Two dosimetric parameters are critical for aPDT outcomes: irradiance (power density, mW/cm^2^) and fluence (energy density, J/cm^2^), where fluence equals irradiance multiplied by exposure time. Most experimental and clinical protocols employ fluences in the ~20–120 J/cm^2^ range, with biofilm eradication generally requiring higher fluences than planktonic cell inactivation. Delivering sub-lethal fluences may not only be ineffective but could also induce stress responses that transiently increase microbial tolerance, underscoring the importance of evidence-based dosimetry [82]. Well-designed aPDT protocols carefully balance PS concentration, incubation time, wavelength, irradiance, and fluence to maximize microbial kill while minimizing damage to host tissues [107].

### 2.4. Phage Therapy

Bacteriophages are highly efficient viruses that selectively infect bacteria. Through the lytic cycle, they bind with remarkable specificity, down to the species and strain level, to surface receptors on the bacterial cell, inject their genome, which then replicates and directs the assembly of structural components, ultimately leading to lysis of the host cell and the release of progeny phages that perpetuate the cycle [108]. A second cycle, the lysogenic pathway, is available only to temperate phages, which inject their genome but remain dormant until an external trigger, such as cellular stress, initiates progression to lysis. In the context of AMR therapies, only the lytic cycle mediated by virulent phages is considered therapeutically relevant, as it represents the most efficient route for bacterial killing [108]. Clinically, bacteriophages are applied in the form of “phage cocktails” for two primary purposes: a cocktail may combine two or more phage strains targeting the same bacterium to reduce the risk of resistance development, or it may combine strains that each target a different bacterial species to broaden the therapeutic range of activity [109]. Administration routes include nebulized inhalation, oral delivery, intravenous injection, and in some cases, bronchoscopy [110]. Although no dedicated framework has been established to evaluate the limitations of each delivery route systematically, available evidence indicates that nebulizers present poor long-term compliance, oral administration carries a high risk of resistance development, intravenous delivery is associated with the risk of antiphage antibody formation, and bronchoscopy may lead to complications such as hypoxia and respiratory depression [110].

Phage therapy (PHT) can also be combined with antibiotics to combat AMR. When a lytic bacteriophage targets the multidrug efflux pumps of an antibiotic-resistant bacterium, the bacterial response to this attack involves modifications to the efflux systems that as a trade-off, decrease resistance and increase susceptibility to several antibiotic classes [111]. In this approach, the lytic phage OMKO1 bound to the outer membrane components of the MexAB and MexXY multidrug efflux systems of *Pseudomonas aeruginosa*, increasing bacterial sensitivity to ceftazidime and ciprofloxacin by twofold and tenfold, respectively [111,112].

Despite these advantages, phage therapy faces limitations that currently constrain its clinical translation. Its high specificity, while reducing off-target effects on the host microbiota, also narrows its spectrum of activity and typically requires strain-level susceptibility testing or the use of tailored, regularly updated phage cocktails to remain effective against evolving bacterial populations [109]. As with antibiotics, bacteria can acquire resistance to phages through mechanisms such as receptor modification, superinfection exclusion, and restriction–modification systems, and this resistance can emerge over the course of treatment [111,112]. Delivery routes carry route-specific risks, including poor long-term compliance with nebulized administration, increased risk of resistance development with oral dosing, antiphage antibody formation with intravenous delivery, and procedural complications such as hypoxia and respiratory depression with bronchoscopy [110]. In addition, the biological nature of phages complicates manufacturing and regulatory approval relative to small-molecule antibiotics, as it requires rigorous quality control, batch-to-batch consistency, and the absence, to date, of an FDA-approved phage therapy product, all of which limit standardization and large-scale clinical adoption [112,113].

### 2.5. Metallic and Metal Oxide Nanoparticles (NPs)

Some metallic nanoparticles exhibit intrinsic antimicrobial activity, as demonstrated for silver nanoparticles (AgNPs), whose effects have been confirmed against several bacterial strains, including *E. coli*, *S. aureus*, and *P. aeruginosa*. Antimicrobial activity has also been reported for copper nanoparticles (CuNPs) [114]. Both CuNPs and AgNPs exert deleterious effects on bacteria without requiring conjugation with other molecules; in contrast, gold nanoparticles (AuNPs) present a high capacity for functionalization, which can further enhance their antimicrobial effects [115]. In general, NPs have reduced dimensions, typically below 100 nm. Due to their small size, they exhibit a high surface area-to-volume ratio and physicochemical properties that differ significantly from those of the same materials at the microscale. For metallic NPs, optical properties are strongly size-dependent. Furthermore, their reduced dimensions confer enhanced mobility through biological tissues.

The primary mechanism of action of metallic nanoparticles involves the release of metal ions and the promotion of hydrogen peroxide (H_2_O_2_) production within cells [116,117]. Although metallic nanoparticles have significant potential for biological and healthcare applications, their toxicity remains an important concern [118]. AgNPs and CuNPs are widely recognized not only for their antimicrobial activity, but also for their toxic potential. Another class of nanoparticles with intrinsic antibacterial activity comprises metal oxide nanoparticles, such as TiO_2_, SiO_2_, MgO, CaO, and ZnO [119]. These materials share mechanisms of action analogous to those of metallic nanoparticles, including metal ion release and the promotion of H_2_O_2_ production, with the addition of some mechanical effects caused by intracellular accumulation [119].

To reduce the working concentrations of metallic nanoparticles, they have been explored as photosensitizers (PSs) in aPDT. In this context, localized surface plasmon resonance (LSPR) has been identified as playing a decisive role in the photoantimicrobial activity of metallic nanoparticles. However, it is not the only relevant effect arising from their interaction with light. LSPR occurs when electromagnetic radiation interacts with metallic nanoparticles that possess a real dielectric constant [120]. These nanostructures contain confined free electrons that oscillate at a characteristic natural frequency. When the frequency of incident electromagnetic radiation matches this natural frequency, resonance occurs between electron oscillations and the radiation’s electric field [120,121]. During LSPR, the amplitude of electron oscillations can promote electron transfer to adjacent molecules, thereby releasing energy as photons and/or heat [116]. These electrons can also be transferred to molecular oxygen, thereby generating ROS.

### 2.6. Antimicrobial Peptides (AMPs)

Antimicrobial peptides (AMPs) are small, naturally occurring molecules that play a central role in the innate immune response of a wide range of organisms, including humans, animals, plants, and microorganisms [122,123]. These peptides serve as a primary defense mechanism against a broad spectrum of pathogens, including bacteria, viruses, fungi, and parasites [122,124]. Their biological importance lies in their ability to neutralize invading microorganisms through diverse mechanisms, including membrane disruption and interference with intracellular processes [122,124,125]. AMPs exhibit significant structural and functional diversity, which allows them to target pathogens through multiple pathways [126,127,128]. In addition to naturally occurring peptides, synthetic and semi-synthetic AMPs have been developed to enhance antimicrobial activity, stability, and specificity, expanding their potential for clinical applications [126,127,128,129].

Gramicidin was the first AMP to be described, discovered in 1939 from *Bacillus brevis* isolated from soil, and has been shown to be effective against bacterial pathogens [124]. At present, numerous AMPs have been cataloged, and various criteria are used to classify them, including origin, structure, biological activity, and amino acid composition [122,123]. Based on origin, AMPs may be categorized as natural or synthetic [122,123]. Natural AMPs are widely distributed across biological kingdoms, including bacteriocins from bacteria, penaeidins and crustins from marine organisms, plant-derived peptides such as defensins and thionins, fungal peptaibols, and mammalian peptides such as defensins, histatins, and LL-37 [122,123,125]. Structurally, AMPs adopt several conformations that are crucial for their function and are commonly divided into four groups: α-helical, β-sheet, mixed α/β structures, and peptides without defined secondary structures [122,123]. α-Helical peptides typically adopt amphipathic conformations that facilitate membrane interaction, whereas β-sheet peptides are stabilized by disulfide bonds, contributing to enhanced structural stability and biological activity [126,127,128]. Regarding antimicrobial spectrum, AMPs can be classified as antibacterial, antifungal, antiviral, or antiparasitic [126,127,128]. Some peptides exhibit broad-spectrum activity, targeting multiple classes of microorganisms [126,127,128,129]. Finally, with respect to amino acid composition, AMPs are often enriched in specific residues, including arginine, histidine, proline, tryptophan, and glycine [122,123]. Most AMPs are cationic and amphipathic, properties that favor interactions with negatively charged microbial membranes, and can be categorized as cationic, anionic, or neutral based on net charge [126,127,128].

AMPs exert antimicrobial effects through two principal mechanisms: membrane disruption and intracellular targeting [122,124,125]. Many AMPs act directly on microbial membranes, leading to structural destabilization through different models, including the toroidal pore, barrel-stave, and carpet mechanisms [124,125]. These interactions result in increased membrane permeability, leakage of intracellular contents, and ultimately cell death [124,125]. In addition, some AMPs penetrate microbial cells and interfere with essential intracellular processes, including the inhibition of DNA and RNA synthesis, interruption of protein synthesis, disruption of enzymatic activity, and compromise of organelle function [124,125]. Some peptides act on both intracellular and extracellular targets and can switch between mechanisms depending on peptide concentration, the target pathogen’s membrane composition, and the organism’s growth phase [124,125].

Despite their therapeutic potential, AMPs face several limitations that hinder their widespread clinical adoption [122,123,126]. A primary challenge is susceptibility to enzymatic degradation, which reduces their stability in biological environments [91,92,127]. Additionally, environmental factors such as pH, ionic strength, and serum components can negatively impact antimicrobial activity [122,123]. Potential cytotoxic effects on mammalian cells must also be considered, as toxicity varies depending on peptide structure, concentration, and route of administration [123,125]. Furthermore, an incomplete understanding of AMP mechanisms of action complicates the rational design of optimized peptides [126,127,128,129,130]. Another significant limitation is the high cost of peptide synthesis and purification, and longer peptides require complex production processes, which can limit scalability and commercial viability [123,125,126]. Although AMPs were initially considered unlikely to induce resistance, several bacterial resistance mechanisms have since been identified [124,126,128]. A common strategy involves modifying the bacterial membrane to reduce its negative charge, thereby decreasing electrostatic attraction to cationic peptides [124,126,128]. Bacteria may also produce biofilms, extracellular matrix components, and outer membrane vesicles that sequester AMPs, preventing them from reaching their targets [125,128]. Additionally, specific enzymes can degrade AMPs, while dedicated transport systems may internalize and inactivate them [125,128]. Collectively, these biological, pharmacokinetic, and economic constraints mean that despite decades of preclinical research, only a small number of AMPs have progressed to advanced clinical trials, underscoring the need for further translational studies before AMPs can be broadly adopted as standalone antimicrobial agents.

### 2.7. Quorum Sensing as a Central Regulator of Virulence

Quorum sensing (QS) represents the central mechanism of bacterial cell–cell communication, enabling coordinated gene expression in response to population density [129,130]. This process depends on the production, release, and detection of signaling molecules known as autoinducers, whose accumulation above a critical threshold triggers synchronized transcriptional responses at the population level. Initially described in bioluminescent Vibrio species, QS is now recognized as a widespread regulatory system controlling key physiological processes, including virulence, biofilm formation, motility, and host–pathogen interactions [129,131]. The consolidation of this field arose from the recognition that bacteria do not behave as isolated unicellular organisms, but rather as structured communities capable of coordination and collective behavior [132,133]. These behaviors are frequently associated with cooperative traits, including the production of public goods and the establishment of biofilm-based lifestyles, reinforcing the concept of functional multicellularity in microbial systems [134,135]. Within this framework, QS-mediated communication enables populations to synchronize gene expressions to maximize collective fitness, despite evolutionary constraints imposed by social cheating and resource sharing [132,134].

At the molecular level, QS systems are highly diverse, encompassing multiple classes of signaling molecules and regulatory circuits. Gram-negative bacteria predominantly employ acyl-homoserine lactones (AHLs), whereas Gram-positive organisms rely on processed oligopeptides; additional universal signals such as autoinducer-2 (AI-2) mediate interspecies communication [130,136]. QS networks are increasingly recognized as key regulators of clinically relevant phenotypes, including biofilm maturation, persistence, and antibiotic tolerance in major human pathogens such as *Pseudomonas aeruginosa* and *Staphylococcus aureus* [131,137]. These systems also integrate environmental and host-derived signals, enabling bacteria to adjust virulence expression dynamically during infection. Importantly, the clinical relevance of QS is supported by evidence obtained directly from patients and from patient-derived bacterial isolates. In a prospective study involving 60 cystic fibrosis patients chronically infected with *Pseudomonas aeruginosa*, quorum-sensing signal molecules were detected in sputum, plasma, and urine and were positively associated with pulmonary bacterial burden. Moreover, plasma concentrations of 2-nonyl-4-hydroxyquinoline (NHQ) were significantly higher during pulmonary exacerbation than during clinical stability, while systemic antibiotic treatment reduced circulating concentrations of selected QS molecules [138]. Complementary evidence has been obtained using the mucoid cystic fibrosis clinical isolate *P. aeruginosa* NH57388A. Quorum-sensing molecules such as N-(3-oxododecanoyl)-L-homoserine lactone (3-oxo-C12-HSL) and the Pseudomonas quinolone signal (PQS), together with supernatants from NH57388A, activate bitter taste receptors expressed by tracheal brush cells. In vivo infection with NH57388A induced TRPM5-dependent airway innate immune responses, including the recruitment of neutrophils, monocytes, and natural killer cells, whereas impaired brush-cell signaling was associated with greater infection severity [139]. Together, these findings demonstrate that QS signaling is active during clinically relevant infection and can directly influence host–pathogen interactions, strengthening the translational rationale for targeting QS as an anti-virulence strategy. Within this conceptual framework, QS has emerged as a strategic target for anti-virulence interventions. Rather than directly eliminating microorganisms, quorum sensing inhibition aims to disrupt the coordinated expression of virulence-associated traits, thereby attenuating pathogenicity and impairing biofilm formation [140,141]. Because these phenotypes are socially regulated and not essential for individual bacterial survival, targeting QS offers a promising alternative to conventional antimicrobial approaches, with the potential to reduce selective pressure for resistance development [142]. However, evolutionary analyses indicate that such strategies may still impose indirect selective pressures and that their efficacy is context-dependent, highlighting the need for careful evaluation of their long-term impact [143]. Anti-virulence targeting is not restricted to quorum-sensing systems. Bacterial protein phosphatases represent another class of regulatory factors that may connect pathogenicity with antimicrobial treatment failure and therefore constitute potential anti-infective targets. In *Mycobacterium tuberculosis*, the secreted protein tyrosine phosphatases PtpA and PtpB promote intracellular survival by interfering with host macrophage signaling. PtpA inhibits phagosome acidification and phagosome–lysosome fusion, whereas PtpB suppresses host antimicrobial and inflammatory responses, making both enzymes attractive targets for therapeutic inhibition [144]. Importantly, this strategy may complement rather than replace conventional antibiotics. Inhibition of MptpB with the small-molecule inhibitor C13 reduced intracellular mycobacterial burden and produced additive effects when combined with rifampicin or bedaquiline, without directly changing extracellular antibiotic susceptibility [145]. In *Staphylococcus aureus*, the eukaryotic-like serine/threonine phosphatase Stp1 provides an even more direct link between virulence and antimicrobial susceptibility. Loss of Stp1 function has been associated with reduced susceptibility to vancomycin, altered cell-wall physiology, and the attenuation of virulence in vivo [146]. Moreover, loss-of-function mutations in *stp1* can promote β-lactam resistance independently of the classical resistance determinants *mecA* and *blaZ* [147]. Together, these findings support the role of bacterial phosphatases as resistance-associated virulence regulators and potential targets for adjunctive anti-infective therapies, although pharmacological inhibition of these enzymes remains predominantly at the preclinical stage. A summary of experimentally observed effects, downstream consequences, and proposed mechanisms by which aPDT may influence bacterial virulence and quorum-sensing-related processes is presented in Table 3.

## 3. Discussion

The increasing spread of antimicrobial resistance has demonstrated that controlling infectious diseases cannot rely solely on the continuous introduction of new antibiotics. However, this scenario does not mean abandoning conventional antimicrobial therapy; rather, it calls for expanding the therapeutic arsenal with complementary strategies that improve treatment efficacy, reduce selective pressure, and overcome limitations associated with resistance, biofilm formation, and persistent infections. In this context, aPDT has emerged as a particularly relevant approach, not as a substitute for antibiotics but as an adjunctive or alternative tool that acts through broad, distinct mechanisms. A defining feature of aPDT within this landscape is its multi-target mechanism: by inducing localized oxidative damage to lipids, proteins, and nucleic acids simultaneously, it is expected to reduce the likelihood that any single resistance mutation confers meaningful protection, a property that, in principle, distinguishes it from approaches relying on a single biochemical interaction, although this expectation is based mainly on short-term experimental studies rather than long-term clinical evidence of resistance-proof behavior.

Natural products have gained considerable attention as antibiotic adjuvants due to their ability to enhance the efficacy of conventional antimicrobials against both susceptible and resistant pathogens. Derived from plants, microorganisms, and marine sources, these bioactive compounds potentiate antibiotic activity through multiple complementary mechanisms. A primary mechanism involves the disruption of bacterial cell membranes, increasing permeability and intracellular drug accumulation. For instance, compounds such as epigallocatechin gallate, myricetin, daidzein, gallic acid, epicatechin, and genistein exhibit synergistic activity against ESKAPE pathogens [155]. Similarly, flavonoids have been shown to enhance the efficacy of tetracycline, erythromycin, and ciprofloxacin [156]. Natural products also target resistance mechanisms by inhibiting efflux pumps, thereby increasing the intracellular antibiotic concentrations (Table 4). For example, baicalein suppresses the NorA efflux system in *S. aureus*, restoring ciprofloxacin activity. In addition, many of these compounds interfere with biofilm formation and quorum sensing, reducing bacterial virulence and improving antibiotic susceptibility. Overall, natural product–antibiotic combinations represent a promising strategy to combat AMR by targeting multiple bacterial pathways simultaneously (Table 4). This approach not only improves therapeutic efficacy, but also reduces selective pressure for the development of resistance, aligning with the “One Health” framework by minimizing impacts on human, animal, and environmental health [142]. In this broader context, it is also important to consider strategies in which natural products are employed not only as antibiotic adjuvants, but also as active components of alternative antimicrobial platforms such as aPDT.

Curcumin has been extensively investigated as a PS for aPDT, demonstrating efficacy against a broad range of bacterial pathogens, including *S. aureus*, MRSA, *S. Typhimurium*, *K. pneumoniae*, and *S. pyogenes*.

In addition, promising antifungal activity has been reported against species such as *C. albicans* (Table 2). These findings highlight the potential of curcumin-based photodynamic approaches as effective alternatives for combating both bacterial and fungal infections. Recent randomized controlled trials have demonstrated that natural PSs such as curcumin, riboflavin, and 5-aminolevulinic acid (5-ALA) can serve as effective adjunctive agents, significantly enhancing microbial reduction when combined with light-based therapies [127]. A summary of commonly investigated natural PSs, along with their sources, activation wavelengths, and antimicrobial efficacy, is presented in Table 2. Thus, compared with natural product–antibiotic combinations alone, natural product-based aPDT offers the added advantage of a light-triggered, multi-target oxidative mechanism that may further enhance antimicrobial efficacy.

The combination of photodynamic therapy and immunotherapy has been proposed as a strategy for treating various cancers [69]. Although this combination has not yet been widely reported in the context of microbial infections, some studies have explored it [165,166]. In these cases, the mechanism of action remains the photooxidative effect on microbial cells, with minimal impact on the surrounding tissue. At the same time, damage to target cells, whether cancerous or bacterial, can release damage-associated molecular patterns (DAMPs), which stimulate immune system activation, including the recruitment of phagocytic cells, promotion of cross-presentation of bacterial antigens to T lymphocytes, increased infiltration of CD4+ and CD8+ T cells, elevated pro-inflammatory cytokines such as TNF-α and IL-1β alongside a decrease in anti-inflammatory cytokines such as IL-10 and TGF-β, and the modulation of macrophage polarization, with an increase in M1 markers such as CD86 and a decrease in M2 markers such as CD206) [69]. In the context of microbial infections, this combination has been studied far less extensively, with only a limited number of reports addressing it directly [165,166]. In these infection-specific studies, the primary mechanism reported remains the direct photooxidative killing of microbial cells, with comparatively minor collateral damage to surrounding tissue, together with more circumscribed evidence of immune involvement: Wang et al. (2023) [165] reported the restoration of leukocyte and lymphocyte counts following macrophage-directed aPDT of MDR bacterial infection, while Zheng et al. (2024) [166] described activation of adaptive cellular and humoral immune responses alongside PDT-mediated bacterial killing in their proposed antimicrobial photodynamic-immune therapy (aPIT) platform. While these findings indicate that aPDT can engage immune mechanisms beyond direct microbial killing, the full DAMP-driven cascade of T-cell recruitment and macrophage repolarization established in cancer PDT has not yet been comprehensively demonstrated in bacterial infection models, and the extent to which it translates to this setting remains to be experimentally established.

These strategies have also been refined by increasing PS selectivity through antibody conjugation, enabling more precise bacterial targeting. For example, the 4497-IgG-IRDye700DX antibody, already approved for use in cancer treatment, has demonstrated a reduction in bacterial load on implant devices colonized with *S. aureus* biofilm in vitro [167]. Although limited to in vitro conditions, the results showed that targeting was restricted to bacteria adjacent to the implant. The IRDye700DX PS is specifically activated by light at the site of infection, generating singlet oxygen and other ROS that promote bacterial death [168]. Similarly, a study by Wang et al. (2023) [165] demonstrated that macrophages can serve as PS carriers, enhancing pathogen specificity through the chemotactic gradient that directs them to the site of infection. In that study, mice with MRSA-infected wounds received macrophages loaded with lysosomal photosensitizers (Lyso700D) activated by light at the infection site. However, the phototoxic effect eliminates not only bacteria in the infected tissue, but also phagocytosed bacteria that are carried away from the wound site as macrophages enter the bloodstream. Results also showed the restoration of immune parameters, with normalization of the lymphocyte and leukocyte levels following treatment [165]. These therapeutic approaches may complement antibiotic therapy by helping to prevent the formation of intracellular bacterial reservoirs, which sustain persistent populations that drive recurrent infection [165,166]. Taken together, these findings reinforce that one of the main strengths of aPDT is not only its direct antimicrobial effect, but also its adaptability through targeted and immune-oriented refinements.

Traditional antibiotic therapy achieves 70.3% clinical success against MDR *P. aeruginosa*, but resistance emerges in 30–53% of cases in nosocomial settings [169,170]. Phage therapy shows more promising clinical outcomes, with an 86.6% success rate in a 16-patient compassionate-use trial of PASA16 [171]. However, resistance development remains possible depending on the susceptibility to the phage used [172]. Conversely, aPDT demonstrates potent in vitro bacterial inactivation at 99–99.9% log reduction without documented resistance development [173], but clinical efficacy data remain limited due to the lack of large-scale clinical trials.

While phage therapy outperforms traditional antibiotics clinically, the mechanisms underlying phage therapy and photodynamic therapy still differ fundamentally in how they interact with bacterial resistance. Bacteriophages actively replicate as living agents within bacterial cells and ultimately cause their rupture. Unlike aPDT, which chemically disrupts multiple cellular targets, such as lipids, proteins, and DNA, without biological interaction with the host cell but requires an external light source, phage therapy depends on successful biological replication within the target organism. However, regarding bacterial resistance, bacteria can develop counter-mechanisms at all stages of the lytic cycle. At the adsorption stage, bacteria may modify or conceal surface receptors, form biofilms that physically restrict phage access, or produce decoy membrane vesicles that sequester phages away from their actual targets. If adsorption is successful, bacteria may still block phage replication through superinfection exclusion (SIE) mediated by prophages, secrete small molecules with anti-phage properties, such as anthracyclines, or employ restriction–modification systems to degrade foreign phage DNA. At the final stage, abortive infection systems can halt replication entirely, with some inducing programmed cell death to prevent the release of progeny phage [174]. In contrast, the development of resistance to aPDT is generally considered more difficult, since ROS act on multiple targets simultaneously, requiring bacteria to evolve protective adaptations against several independent forms of oxidative damage at once. It is important to note, however, that this assumption is based primarily on the mechanistic rationale of multi-target oxidative damage and on a still-limited number of experimental studies, most of which were conducted under short-term or single-exposure conditions. Current investigations of bacterial resistance to aPDT have reported limited evidence of stable, heritable resistance; nonetheless, phenomena such as sublethal stress adaptation and transient tolerance have already been documented, and given the continuous adaptive capacity of microorganisms under repeated or sublethal exposure, the possibility of resistance or reduced susceptibility emerging over time cannot be definitively excluded. Long-term, clinically relevant studies are still needed to confirm whether this theoretical resistance-resilience advantage is maintained under real-world treatment conditions [83]. In this respect, aPDT and phage therapy present a complementary risk profile: phages offer unmatched precision but face a defined set of bacterial counter-strategies at each lytic stage, while aPDT’s multi-target oxidative action makes the emergence of stable resistance less straightforward, though neither strategy is immune to adaptation by sufficiently plastic bacterial populations.

Bacteriophages are also highly strain-specific [108], targeting precise surface receptors that may vary even among strains of the same species. While this specificity makes them precise therapeutic agents with minimal disruption to the host’s commensal microbiota, it also presents a clinical challenge: accurate pathogen identification is required before treatment, particularly in polymicrobial infections involving multiple bacterial species. This may necessitate tailored phage cocktails [109], adding complexity and cost to the therapeutic process. Moreover, the emergence of resistance during phage therapy represents an ongoing clinical constraint, as detailed earlier in this section, reducing phage efficacy over the course of treatment. Additionally, phage therapy products face standardization challenges in manufacturing and quality control; unlike chemically synthesized photosensitizers or antibiotics, batch-to-batch variability in phage preparations can compromise consistency and reproducibility in clinical settings, complicating regulatory pathways for clinical translation [175]. PSs used in aPDT have a broader spectrum, capable of inactivating a wider range of Gram-positive and Gram-negative bacteria without prior pathogen identification. However, this broader action also means that ROS generation may affect the surrounding host tissues. This limitation is particularly relevant in systemic infections. In this context, the choice of photosensitizer and light-delivery system is a key factor influencing the clinical photodynamic activity [176]. One potential approach is the development of photosensitizers that are metabolized and/or eliminated more rapidly by healthy cells than by pathogenic cells or photosensitizers that are selective to pathogenic cells [176]. In parallel, targeted light delivery may represent another strategy to improve the safety of aPDT [176]. Furthermore, it is important to consider the anatomical location of the infection when selecting the appropriate PS/light pair [176]. Despite these limitations, when rapid intervention or the control of polymicrobial infections is required, the broader action of aPDT may represent a practical advantage.

A compelling demonstration of the phage–PDT combination was presented by Petrosino et al. (2023), who engineered the M13 bacteriophage for targeted photodynamic killing [167]. This approach addresses one of aPDT’s major limitations, collateral damage to non-target cells, by exploiting the strain specificity of bacteriophages as a precision delivery system for PSs [167]. The phage was functionalized with rose bengal and displayed peptides targeting Gram-negative pathogens; upon light irradiation, the targeted bacteria were killed at subnanomolar concentrations, highlighting the potency of the synergy between phage therapy and PDT oxidative mechanisms. This represents a promising frontier in which the complementary strengths of both therapies can be preserved, with each approach mitigating the other’s limitations [167]. From a regulatory standpoint, the comparison is more nuanced. While photosensitizers have received FDA approval for various clinical applications in oncologic diseases, as well as dermatologic, ophthalmologic, cardiovascular, and other areas [177], approval specifically for aPDT as an antimicrobial treatment requires separate regulatory validation and is not yet established as of 2026. Bacteriophages, as biological agents, face additional regulatory hurdles: as of 2026, no phage therapy product has received FDA approval for clinical antimicrobial use, though several compassionate-use programs are active [178]. Thus, neither approach currently holds a clear regulatory advantage for antimicrobial deployment.

Different classes of antimicrobial agents have been associated with distinct nanoparticle systems to help mitigate AMR. Notable examples include the combination of amoxicillin or ampicillin with AgNPs, ciprofloxacin with zinc oxide nanoparticles, and vancomycin-conjugated AuNPs [179]. In addition to metallic nanoparticles, lipid-based nanocarriers, particularly liposomal nanoparticles (LNPs), have garnered significant attention. Engineered as delivery systems for antimicrobial agents, LNPs consist of phospholipid bilayers surrounding an aqueous core, enabling the encapsulation of both hydrophilic and hydrophobic compounds [180]. LNPs offer several advantages, including the protection of encapsulated drugs from degradation, as well as controlled and targeted drug release. Consequently, they have been widely employed to deliver agents such as vancomycin, colistin, and amphotericin B, particularly for the treatment of resistant bacterial and fungal infections [180]. At the same time, the main advantage of nanoparticles may also be one of their primary limitations: particles composed of the same material can exhibit significantly different outcomes in terms of efficacy and toxicity depending on their size and shape. In this context, the combination of nanoparticles with aPDT emerges as a promising strategy, enabling lower nanoparticle concentrations while still achieving effective bacterial inactivation and potentially reducing systemic toxicity [180]. This connection is especially relevant because it positions aPDT not in opposition to nanotechnology, but as a platform that may benefit from and strengthen nanoparticle-based delivery systems [180].

It is important to emphasize that AMR encompasses a wide range of mechanisms, including enzymatic degradation of drugs, target modification, alterations in membrane permeability, and efflux pump activity [21,181]. In this context, nanoparticle–antibiotic combinations have demonstrated synergistic and/or antagonistic effects against different microorganisms. In many cases, however, the mechanisms underlying these effects have not been empirically elucidated [179].

In the literature, the synergistic effect observed with zinc oxide nanoparticles combined with ciprofloxacin has been associated with inhibition of the NorA efflux pump, thereby reducing ciprofloxacin extrusion. In this case, the combination appears to interfere with a specific bacterial resistance mechanism against ciprofloxacin [182]. Similarly, gold nanoparticles (AuNPs) conjugated with vancomycin appear to enhance their interaction with peptide components present on the bacterial cell surface, promoting disruption of the biological membrane, as expected from vancomycin activity [183,184].

Furthermore, silver nanoparticles (AgNPs) have demonstrated synergistic effects with several classes of antimicrobial agents against different bacterial species [185]. In this case, the synergistic effect has been attributed to enhanced antimicrobial permeabilization resulting from specific interactions between the nanoparticles and components of the plasma membrane. Thus, in the examples described above, synergism has been attributed either to the proposed mechanisms, or in some cases, supported by empirical evidence, suggesting that nanoparticles may attenuate and/or inhibit specific bacterial resistance mechanisms, such as efflux pump inhibition and increased membrane permeability, thereby facilitating drug internalization [186,187]. Nevertheless, it is important to emphasize that the scientific literature also includes studies demonstrating the development of resistance to AgNPs. In *E. coli* strains, phenotypic alterations have been reported that promote nanoparticle aggregation, consequently reducing their antimicrobial activity [188].

Conversely, within the context of aPDT, molecules with low intrinsic toxicity can be employed while maintaining significant photodynamic efficacy. Furthermore, *E. coli* and *S. aureus* strains did not develop resistance to photodynamic therapy, even after 20 cycles of treatment [189].

In this regard, aPDT represents a promising alternative, both due to the potential use of less toxic photosensitizers (PSs) and its ability to act on multiple cellular targets [71]. No strong evidence of resistance has developed to aPDT therapy [189]. Therefore, when comparing aPDT with nanoparticle-based strategies, it is essential to carefully evaluate the balance between potential risks and efficacy, as well as the potential benefits of combining both approaches to minimize the associated risks.

The combination of AMPs with aPDT has also emerged as a promising strategy to enhance antimicrobial efficacy [173,174]. In this approach, AMPs are often conjugated to PSs, improving their solubility and facilitating interaction with microbial cells [190,191]. The advantage of this combined therapy lies in complementary mechanisms of action: AMPs promote membrane disruption and facilitate PS uptake. At the same time, aPDT enhances ROS production, thereby inducing oxidative damage to cellular components [173,174]. Significant reductions in microbial load have been demonstrated when AMPs are combined with aPDT, including against *S. aureus*, *E. coli*, and *P. aeruginosa* [190,192]. These studies employed a variety of PSs, such as protoporphyrin IX, porphyrin, temoporfin, methylene blue, chlorin e6, rose bengal, and phthalocyanine [190,192]. Beyond effectiveness against planktonic cells, this combined approach has also shown promising results against biofilms [173,174]. This strategy once again underscores a central advantage of aPDT: its ability to integrate with other antimicrobial platforms while preserving its broad oxidative, multi-target action [190,193].

Antimicrobial photodynamic therapy has been traditionally described as a bactericidal approach based on ROS generation following PS activation by light in the presence of oxygen [74,80]. However, experimental studies indicate that aPDT, particularly under sublethal exposure conditions, can alter QS-associated gene expression and virulence-related phenotypes. These experimentally observed effects should be distinguished from a defined QS-specific mechanism of aPDT. In *Pseudomonas aeruginosa*, ALA-PDT has been shown to reduce the expression of QS-related genes, as well as pyocyanin and elastase secretion [148]. In contrast, sublethal methylene-blue-mediated photodynamic inactivation downregulated *lasI*, *lasR*, *rhlI*, *rhlR*, and *rhlA* while increasing *phzM* expression and pyocyanin production in some strains [149]. Other sublethal protocols have produced increased activity of the Las QS system while leaving *rhl*- and *pqs*-associated responses largely unchanged [150]. Thus, the modulation of QS and virulence-associated phenotypes is directly supported by experimental evidence, but the direction of these responses is treatment- and context-dependent and should not be interpreted as evidence for a single, uniformly inhibitory anti-virulence mechanism. Biofilm disruption is likewise a directly demonstrated effect of aPDT across multiple experimental models [78,151]. However, reduced biofilm biomass and matrix destabilization may result from a combination of microbial killing, oxidative damage to extracellular and cellular components, and altered regulatory signaling. Therefore, biofilm disruption should be regarded primarily as an experimentally observed outcome of photodynamic treatment rather than as evidence of a specific QS-targeted mechanism. In contrast, direct ROS-mediated interference with bacterial regulatory proteins or QS signaling molecules remains a proposed mechanistic explanation. Oxidative stress is known to modify proteins and regulatory networks [152,153], but direct evidence demonstrating that aPDT specifically oxidizes LuxR-type regulators or quorum-sensing molecules in situ remains limited. Such processes should therefore be interpreted as plausible explanations for the observed transcriptional and phenotypic responses rather than established primary mechanisms of aPDT. Similarly, persistent phenotypic changes following sublethal exposure are better understood as downstream, stress-associated adaptive responses rather than primary mechanisms of photodynamic action.

Biofilm formation, a process tightly regulated by QS, is a major determinant of bacterial persistence and antimicrobial tolerance. aPDT has demonstrated significant efficacy in disrupting biofilm structure and inhibiting its formation across multiple pathogens [78,151]. Importantly, the effects of aPDT extend beyond biomass reduction, affecting extracellular matrix integrity and impairing intercellular communication within the biofilm [78,151]. This disruption compromises the coordinated behavior necessary for biofilm maintenance and resilience, particularly in chronic infections where biofilms play a central protective role. Therefore, in addition to killing planktonic cells, aPDT may weaken one of the major protective strategies bacteria employ during persistent infection. The biological effects of aPDT are primarily mediated by ROS, including singlet oxygen and free radicals, which induce oxidative damage to cellular components [78]. Beyond cytotoxicity, however, ROS can also modulate bacterial signaling pathways, affecting gene expression and the function of regulatory proteins [152]. Oxidative modifications of transcriptional regulators, including LuxR-type proteins, may alter their ability to bind autoinducers or DNA, thereby disrupting QS signaling [152]. Additionally, ROS may affect the stability and diffusion of signaling molecules themselves, further compromising communication networks and coordinated virulence responses. This mechanistic perspective helps explain why aPDT may influence not only bacterial survival, but also bacterial behavior [152]. Recent studies have also highlighted that sublethal photodynamic stress can induce persistent phenotypic changes in bacterial populations, including alterations in gene expression related to virulence and stress-response pathways [154]. In this context, the term “phenotypic memory” refers to transient, non-heritable changes in gene expression or physiological state that persist for a limited period after the initial stress exposure and can influence how bacterial cells respond to subsequent environmental challenges; it does not imply a stable or heritable trait. This phenomenon should therefore be clearly distinguished from classical antimicrobial resistance, which arises from heritable genetic mutations or horizontally acquired resistance determinants that are stably transmitted to daughter cells regardless of continued exposure to the selective agent. Phenotypic memory, in contrast, is expected to fade over subsequent generations in the absence of repeated stress, although the duration and stability of this adaptive state following sublethal aPDT exposure have not yet been systematically characterized. With this distinction in mind, sublethal photodynamic stress may still be relevant to aPDT’s potential as an anti-virulence strategy, as it raises the possibility of the transient attenuation of pathogenic traits without the heritable, stable changes that define classical resistance mechanisms [154,194,195]. Despite its potential, the application of aPDT as an anti-virulence strategy presents important limitations. The biological response is highly dose-dependent: higher doses lead to bactericidal effects that may reintroduce selective pressure like that of conventional antimicrobials [196]. Furthermore, the impact of aPDT on virulence modulation may vary across species, strains, and environmental conditions, reflecting differences in oxidative stress tolerance and regulatory network architecture. Another critical limitation is the scarcity of clinical studies validating these effects in vivo, which currently restricts the translational application of aPDT as an anti-virulence strategy. Even so, these limitations do not diminish the broader relevance of aPDT; rather, they highlight the need for more refined protocols and stronger clinical validation to fully exploit its multifunctional antimicrobial potential.

## 4. Conclusions

In summary, the evidence presented throughout this review indicates that aPDT represents an important addition to current antimicrobial strategies in the era of resistance. Each modality examined, natural products, immunotherapy, phage therapy, nanoparticles, antimicrobial peptides, and anti-virulence strategies, demonstrates that bacterial resistance can and must be countered through diverse, complementary mechanisms rather than a single approach. Within this broader therapeutic landscape, aPDT offers something most resistance-targeting strategies lack: a mechanism that bacteria cannot easily evolve around, applicability at the point of infection, and compatibility with the pharmacological arsenal we already have. The aPDT demonstrated compatibility with antibiotics, immunotherapy, nanoparticles, and antimicrobial peptides, positioning it not as a replacement but as a force multiplier for therapies already in clinical use. The question is no longer whether complementary strategies can help; it is whether the clinical and regulatory infrastructure will move quickly enough to implement them. Antibiotics are not obsolete. But used in isolation, they soon will be.

## Figures and Tables

**Table 2:** Natural photosensitizers for antimicrobial photodynamic therapy.

NP-PS	PDT Parameters	Microorganism/Activity	Reference
Curcumin (CUR)	2.5 µM CUR, light dose (LD) 6.4 J/cm^2^ 450 nm.	Enhances the conventional antibiotics’ activity against *S. pneumoniae* and *S. pyogenes*	[95]
	1 μM CUR and LD 2.5 J/cm^2^, 450 nm	Enhances activity against *S. aureus*	[96]
	2.5 µM, 450 nm, and LD 15 J/cm^2^	Enhanced antifungal efficacy against *C. albicans*	[97]
	CUR 10 µM, LD 10 J/cm^2^	Potentiate the antibiotics amoxicillin, erythromycin, and gentamicin’s effect against *S. aureus*.	[98]
Riboflavin (RF)	0.1% RF, 445 nm, LD 1.24 J/cm^2^	Inhibits *S. aureus* and *C. albicans*	[99]
Aloe-emodin (AE)	AE 0.5 to 100 µM, 435 nm, 80 mW/cm^2^, 10–40 min	Light and AE dose-dependent inhibition of *P. aeruginosa*	[100]
Caffeic acid (CA)	3 mM CA, 400 nm, 3–5 J/cm^2^	Damage the cell membranes of *E. coli*, *S. enterica*, and *L. monocytogenes*	[101]
Hypocrellin B	470 nm, 0.7 J/cm^2^	Inhibits *S. aureus*	[102]
Farnesol	0.25 mM, 660 nm	Inhibits *E. faecalis*	[103]

**Table 3:** Experimentally observed effects, downstream consequences, and proposed mechanisms associated with the influence of aPDT on bacterial virulence and quorum-sensing-related processes.

Process/Target	Reported Effect	Evidence Status/Interpretation	Microorganism	References
QS modulation	Altered expression of QS-regulated genes, including *las* and *rhl* systems; the direction and magnitude of the response depend on treatment conditions	Direct experimental evidence from gene-expression and reporter assays; response is protocol- and dose-dependent and should not be interpreted as uniformly inhibitory	*Pseudomonas aeruginosa*	[148,149,150]
Virulence-associated traits	Changes in pyocyanin, elastase, rhamnolipid, protease, and other QS-regulated phenotypes	Direct experimental evidence at the phenotypic and/or transcriptional level; represents an observed outcome of photodynamic stress rather than proof of a specific anti-virulence mechanism	*P. aeruginosa* and other bacterial models	[148,149]
Biofilm disruption	Reduced biofilm biomass, structural damage, and extracellular-matrix destabilization	Directly demonstrated outcome in experimental models; likely reflects combined microbial killing, oxidative matrix damage, and potentially altered regulatory signaling rather than a single QS-specific mechanism	*P. aeruginosa*, *S. aureus*, and mixed biofilms	[78,148,151]
ROS-mediated signaling interference	Oxidative modification of regulatory proteins and perturbation of signaling pathways	Proposed mechanistic explanation largely inferred from general oxidative-stress biology; direct demonstration as a QS-specific mechanism of aPDT remains limited	General bacterial systems	[152,153]
Phenotypic adaptation following sublethal aPDT	Persistent or delayed changes in stress-response and virulence-associated phenotypes or gene expression	Emerging downstream adaptive response should not be considered an established primary mechanism of aPDT or equivalent to classical antimicrobial resistance	*S. aureus* and other experimental bacterial models	[154]

**Table 4:** Natural products as antibiotic adjuvants.

NP Compound	Antibiotic	Antimicrobial Activity	Reference
Curcumin	Amikacin (AMK), Moxifloxacin (MOX)	Synergy with AMK and MOX against *M. tuberculosis*	[157]
Berberine	Rifaximin	Synergistic effect against *K. pneumoniae*	[158]
	Linezolid, Cefoxitin, and Erythromycin	Enhances antibiotic activity against *Staphylococcus* spp.	[159]
Resveratrol	Polymyxin B	Enhances activity against *K. pneumoniae* and *E. coli*	[160]
Quercetin	Colistin	Synergistic activity against *A. baumannii*	[161]
Baicalein	Rifampicin	Synergistic activity against *S. aureus* biofilms	[162]
Kuwanon G	Oxacillin or Gentamicin	Synergistic activity against MRSA	[163]
Citral	Norfloxacin	Synergistic activity against MRSA	[164]

## Data Availability

No new data were created or analyzed in this study. Data sharing is not applicable to this article.
